# Supplementary material for: Acute disruption of the synaptic vesicle membrane protein synaptotagmin 1 using knockoff in mouse hippocampal neurons
Source: eLife. 2020 Jun 9;9:e56469. doi: 10.7554/eLife.56469 (PMC7282819; doi:10.7554/eLife.56469)
Supplement: Figure 5—source data 1. — Table summarizing the Kruskal-Wallis test and Dunn’s multiple comparison test for the histograms shown in Figure 5b. [file elife-56469-fig5-data1.docx]

**Figure 5-source data 1**

| **Group** | **Mean** | **SEM** | **N** |  |
| --- | --- | --- | --- | --- |
| Wild type (*Syt1* fl/fl) | 53.31 | 0.3131 | 1655 |  |
| *Syt1* KO (+CRE) | 67.04 | 1.132 | 584 |  |
| S1KO +S1-SELF +0.5 μM PRV | 55.41 | 0.8579 | 518 |  |
| S1-SELF 2h washout | 55.28 | 0.4648 | 759 |  |
| S1-SELF 4h washout | 64.06 | 1.753 | 221 |  |
| S1-SELF 6h washout | 61.96 | 1.049 | 565 |  |
| S1-SELF 8h washout | 66.73 | 2.046 | 183 |  |
|  |  |  |  |  |
| **Dunn's multiple comparisons test** | **Mean rank diff.** | **Significant?** | **Summary** | **Adjusted P Value** |
| Wild type (*Syt1* fl/fl) vs. *Syt1* KO (+CRE) | -1637 | Yes | **** | <0.0001 |
| Wild type (*Syt1* fl/fl) vs. S1KO +S1-SELF +0.5 μM PRV | -103.7 | No | ns | >0.9999 |
| Wild type (*Syt1* fl/fl) vs. S1-SELF 2h washout | -791.8 | Yes | **** | <0.0001 |
| Wild type (*Syt1* fl/fl) vs. S1-SELF 4h washout | -1224 | Yes | **** | <0.0001 |
| Wild type (*Syt1* fl/fl) vs. S1-SELF 6h washout | -1235 | Yes | **** | <0.0001 |
| Wild type (*Syt1* fl/fl) vs. S1-SELF 8h washout | -1477 | Yes | **** | <0.0001 |
| *Syt1* KO (+CRE) vs. S1KO +S1-SELF +0.5 μM PRV | 1533 | Yes | **** | <0.0001 |
| *Syt1* KO (+CRE) vs. S1-SELF 2h washout | 845 | Yes | **** | <0.0001 |
| *Syt1* KO (+CRE) vs. S1-SELF 4h washout | 412.7 | Yes | *** | 0.0006 |
| *Syt1* KO (+CRE) vs. S1-SELF 6h washout | 401.6 | Yes | **** | <0.0001 |
| *Syt1* KO (+CRE) vs. S1-SELF 8h washout | 160.3 | No | ns | >0.9999 |
| S1KO +S1-SELF +0.5 μM PRV vs. S1-SELF 2h washout | -688.2 | Yes | **** | <0.0001 |
| S1KO +S1-SELF +0.5 μM PRV vs. S1-SELF 4h washout | -1120 | Yes | **** | <0.0001 |
| S1KO +S1-SELF +0.5 μM PRV vs. S1-SELF 6h washout | -1132 | Yes | **** | <0.0001 |
| S1KO +S1-SELF +0.5 μM PRV vs. S1-SELF 8h washout | -1373 | Yes | **** | <0.0001 |
| S1-SELF 2h washout vs. S1-SELF 4h washout | -432.3 | Yes | *** | 0.0001 |
| S1-SELF 2h washout vs. S1-SELF 6h washout | -443.4 | Yes | **** | <0.0001 |
| S1-SELF 2h washout vs. S1-SELF 8h washout | -684.7 | Yes | **** | <0.0001 |
| S1-SELF 4h washout vs. S1-SELF 6h washout | -11.1 | No | ns | >0.9999 |
| S1-SELF 4h washout vs. S1-SELF 8h washout | -252.4 | No | ns | 0.9054 |
| S1-SELF 6h washout vs. S1-SELF 8h washout | -241.3 | No | ns | 0.4846 |
