# Supplementary material for: Acute disruption of the synaptic vesicle membrane protein synaptotagmin 1 using knockoff in mouse hippocampal neurons
Source: eLife. 2020 Jun 9;9:e56469. doi: 10.7554/eLife.56469 (PMC7282819; doi:10.7554/eLife.56469)
Supplement: Figure 6—source data 1. — Table summarizing the Kruskal-Wallis test and Dunn’s multiple comparison test for data in Figure 6a. [file elife-56469-fig6-data1.docx]

**Figure 6-source data 1**

| **Group** | **Mean** | **SEM** | **N** |  |
| --- | --- | --- | --- | --- |
| Wild type (*Syt1* fl/fl) | 2.212 | 0.4706 | 12 |  |
| *Syt1* KO (+CRE) | 7.791 | 1.407 | 16 |  |
| S1KO +S1-SELF +0.5 μM PRV | 3.713 | 0.5313 | 16 |  |
| S1-SELF 4h washout | 9.333 | 1.375 | 18 |  |
|  |  |  |  |  |
| **Dunn's multiple comparisons test** | **Mean rank diff.** | **Significant?** | **Summary** | **Adjusted P Value** |
| WT vs. S1KO | -23.17 | Yes | ** | 0.0046 |
| WT vs. S1KO + S1-SELF +PRV | -9.135 | No | ns | >0,9999 |
| WT vs. S1KO +S1-SELF -4h PRV | -28.69 | Yes | *** | 0.0001 |
| S1KO vs. S1KO + S1-SELF +PRV | 14.03 | No | ns | 0.1669 |
| S1KO vs. S1KO +S1-SELF -4h PRV | -5.528 | No | ns | >0,9999 |
| S1KO + S1-SELF +PRV vs. S1KO +S1-SELF -4h PRV | -19.56 | Yes | ** | 0.0096 |
