# Supplementary material for: Acute disruption of the synaptic vesicle membrane protein synaptotagmin 1 using knockoff in mouse hippocampal neurons
Source: eLife. 2020 Jun 9;9:e56469. doi: 10.7554/eLife.56469 (PMC7282819; doi:10.7554/eLife.56469)
Supplement: Figure 6—source data 2. — Table summarizing the Kruskal-Wallis test and Dunn’s multiple comparison test for data in Figure 6f. [file elife-56469-fig6-data2.docx]

**Figure 6-source data 2**

| **Group** | **Mean** | **SEM** | **N** |  |
| --- | --- | --- | --- | --- |
| Wild type (*Syt1* fl/fl) | 0.3914 | 0.04079 | 21 |  |
| *Syt1* KO (+CRE) | 0.9367 | 0.1335 | 18 |  |
| S1KO +S1-SELF +0.5 μM PRV | 0.3331 | 0.02947 | 13 |  |
| S1-SELF 2h washout | 0.6533 | 0.08528 | 15 |  |
| S1-SELF 4h washout | 1.201 | 0.1758 | 13 |  |
| S1-SELF 6h washout | 1.127 | 0.2118 | 15 |  |
| S1-SELF 8h washout | 1.196 | 0.3175 | 17 |  |
|  |  |  |  |  |
| **Dunn's multiple comparisons test** | **Mean rank diff.** | **Significant?** | **Summary** | **Adjusted P Value** |
| Wild type (*Syt1* fl/fl) vs. *Syt1* KO (+CRE) | -43.14 | Yes | *** | 0.0007 |
| Wild type (*Syt1* fl/fl) vs. S1KO +S1-SELF +0.5 μM PRV | 6.524 | No | ns | >0.9999 |
| Wild type (*Syt1* fl/fl) vs. S1-SELF 2h washout | -24.58 | No | ns | 0.5278 |
| Wild type (*Syt1* fl/fl) vs. S1-SELF 4h washout | -53.78 | Yes | **** | <0.0001 |
| Wild type (*Syt1* fl/fl) vs. S1-SELF 6h washout | -43.98 | Yes | ** | 0.0013 |
| Wild type (*Syt1* fl/fl) vs. S1-SELF 8h washout | -45.3 | Yes | *** | 0.0004 |
| *Syt1* KO (+CRE) vs. S1KO +S1-SELF +0.5 μM PRV | 49.67 | Yes | *** | 0.0006 |
| *Syt1* KO (+CRE) vs. S1-SELF 2h washout | 18.57 | No | ns | >0.9999 |
| *Syt1* KO (+CRE) vs. S1-SELF 4h washout | -10.64 | No | ns | >0.9999 |
| *Syt1* KO (+CRE) vs. S1-SELF 6h washout | -0.8333 | No | ns | >0.9999 |
| *Syt1* KO (+CRE) vs. S1-SELF 8h washout | -2.157 | No | ns | >0.9999 |
| S1KO +S1-SELF +0.5 μM PRV vs. S1-SELF 2h washout | -31.1 | No | ns | 0.2408 |
| S1KO +S1-SELF +0.5 μM PRV vs. S1-SELF 4h washout | -60.31 | Yes | **** | <0.0001 |
| S1KO +S1-SELF +0.5 μM PRV vs. S1-SELF 6h washout | -50.5 | Yes | *** | 0.0008 |
| S1KO +S1-SELF +0.5 μM PRV vs. S1-SELF 8h washout | -51.82 | Yes | *** | 0.0003 |
| S1-SELF 2h washout vs. S1-SELF 4h washout | -29.21 | No | ns | 0.3692 |
| S1-SELF 2h washout vs. S1-SELF 6h washout | -19.4 | No | ns | >0.9999 |
| S1-SELF 2h washout vs. S1-SELF 8h washout | -20.72 | No | ns | >0.9999 |
| S1-SELF 4h washout vs. S1-SELF 6h washout | 9.808 | No | ns | >0.9999 |
| S1-SELF 4h washout vs. S1-SELF 8h washout | 8.484 | No | ns | >0.9999 |
| S1-SELF 6h washout vs. S1-SELF 8h washout | -1.324 | No | ns | >0.9999 |
