# Supplementary material for: Acute disruption of the synaptic vesicle membrane protein synaptotagmin 1 using knockoff in mouse hippocampal neurons
Source: eLife. 2020 Jun 9;9:e56469. doi: 10.7554/eLife.56469 (PMC7282819; doi:10.7554/eLife.56469)
Supplement: Figure 6—source data 3. — Table summarizing the Kruskal-Wallis test and Dunn’s multiple comparison test for histogram data in Figure 6g. [file elife-56469-fig6-data3.docx]

**Figure 6-source data 3**

| **Group** | **Mean** | **SEM** | **N** |  |
| --- | --- | --- | --- | --- |
| Wild type (*Syt1* fl/fl) | 67.91 | 5.325 | 15 |  |
| *Syt1* KO (+CRE) | 5.765 | 1.928 | 15 |  |
| S1KO +S1-SELF +0.5 μM PRV | 62.21 | 3.979 | 16 |  |
| S1-SELF 2h washout | 39.83 | 4.751 | 15 |  |
| S1-SELF 4h washout | 27.6 | 6.768 | 11 |  |
| S1-SELF 6h washout | 18.92 | 4.694 | 15 |  |
| S1-SELF 8h washout | 13.33 | 2.677 | 10 |  |
|  |  |  |  |  |
| **Dunn's multiple comparisons test** | **Mean rank diff.** | **Significant?** | **Summary** | **Adjusted P Value** |
| Wild type (*Syt1* fl/fl) vs. *Syt1* KO (+CRE) | 64.43 | Yes | **** | <0.0001 |
| Wild type (*Syt1* fl/fl) vs. S1KO +S1-SELF +0.5 μM PRV | 4.973 | No | ns | >0.9999 |
| Wild type (*Syt1* fl/fl) vs. S1-SELF 2h washout | 24.03 | No | ns | 0.4061 |
| Wild type (*Syt1* fl/fl) vs. S1-SELF 4h washout | 36.88 | Yes | * | 0.0202 |
| Wild type (*Syt1* fl/fl) vs. S1-SELF 6h washout | 46.6 | Yes | *** | 0.0001 |
| Wild type (*Syt1* fl/fl) vs. S1-SELF 8h washout | 50.22 | Yes | *** | 0.0003 |
| *Syt1* KO (+CRE) vs. S1KO +S1-SELF +0.5 μM PRV | -59.46 | Yes | **** | <0.0001 |
| *Syt1* KO (+CRE) vs. S1-SELF 2h washout | -40.4 | Yes | ** | 0.0018 |
| *Syt1* KO (+CRE) vs. S1-SELF 4h washout | -27.55 | No | ns | 0.2868 |
| *Syt1* KO (+CRE) vs. S1-SELF 6h washout | -17.83 | No | ns | >0.9999 |
| *Syt1* KO (+CRE) vs. S1-SELF 8h washout | -14.22 | No | ns | >0.9999 |
| S1KO +S1-SELF +0.5 μM PRV vs. S1-SELF 2h washout | 19.06 | No | ns | >0.9999 |
| S1KO +S1-SELF +0.5 μM PRV vs. S1-SELF 4h washout | 31.91 | No | ns | 0.0795 |
| S1KO +S1-SELF +0.5 μM PRV vs. S1-SELF 6h washout | 41.63 | Yes | *** | 0.0008 |
| S1KO +S1-SELF +0.5 μM PRV vs. S1-SELF 8h washout | 45.24 | Yes | ** | 0.0014 |
| S1-SELF 2h washout vs. S1-SELF 4h washout | 12.85 | No | ns | >0.9999 |
| S1-SELF 2h washout vs. S1-SELF 6h washout | 22.57 | No | ns | 0.5897 |
| S1-SELF 2h washout vs. S1-SELF 8h washout | 26.18 | No | ns | 0.4758 |
| S1-SELF 4h washout vs. S1-SELF 6h washout | 9.715 | No | ns | >0.9999 |
| S1-SELF 4h washout vs. S1-SELF 8h washout | 13.33 | No | ns | >0.9999 |
| S1-SELF 6h washout vs. S1-SELF 8h washout | 3.617 | No | ns | >0.9999 |
