# Supplementary material for: Acute disruption of the synaptic vesicle membrane protein synaptotagmin 1 using knockoff in mouse hippocampal neurons
Source: eLife. 2020 Jun 9;9:e56469. doi: 10.7554/eLife.56469 (PMC7282819; doi:10.7554/eLife.56469)
Supplement: Supplementary file 1. — Table summarizing commercially available HCV inhibitors and their properties. [file elife-56469-supp1.docx]

**Supplementary File 1**

| Inhibitor | Abbr. | IC_50_ | EC_50_ | CC_50_ | Cell type | Covalent (Y/N) | Type |
| --- | --- | --- | --- | --- | --- | --- | --- |
| Ciluprevir  (BILN-2061) | CLV |  | 4.0 nM | 33 µM | HuH-7 | N | P1/P3 macrocyclic |
| Asunaprevir  (BMS-650032) | ASV | 0.7 nM | 4.0 nM | 11 µM  19 µM | HEK293  HeLa | N | Tripeptidic acylsulfonamide |
| Simeprevir (TMC435) | SMV |  | 28 nM | >42 µM  47 µM | HEK293  HuH-7 | N | P1/P3 macrocyclic  acylsulfonamide |
| Grazoprevir  (MK-5172) | GZV | 0.01 nM | 0.37 - 2.0 nM | >50 µM | HuH-7 | N | P2/P4 macrocyclic  acylsulfonamide |
| Danoprevir  (ITMN-191) | DNV | 0.2 nM | 1.6 nM (1b) | >75 µM | Various | Semi-covalent | P1/P3 macrocyclic  acylsulfonamide |
| Paritaprevir  (ABT-450) | PRV | 0.18 nM | 1.0 nM | >37 µM | not stated | N | P1/P3 macrocyclic  acylsulfonamide |
| Glecaprevir  (ABT-493) | GCV | 4.6 nM | 0.85 nM | 72 µM  62 µM  59 µM | Huh-7  HepG2  MT4 | N | P2/P4 macrocyclic  acylsulfonamide |
